# The role and research progress of the MET signalling pathway in targeted therapy for osteosarcoma

**DOI:** 10.3389/or.2025.1615111

**Published:** 2025-06-17

**Authors:** Qilong Su, Jingyu Hou, Xinhui Du, Fan Zhang, Panhong Zhang, Bangmin Wang, Weitao Yao

**Affiliations:** The Affiliated Cancer Hospital of Zhengzhou University & Henan Cancer Hospital, Zhengzhou, China

**Keywords:** osteosarcoma, MET signalling pathway, targeted therapy, small molecule inhibitors, specific antibody, combination therapy

## Abstract

MET, a receptor tyrosine kinase proto-oncogene and the specific receptor for hepatocyte growth factor (HGF), plays a critical role in the initiation and progression of osteosarcoma (OS) through sustained pathway activation. Aberrant activation of MET has been shown to trigger multiple downstream signalling pathways, including RAS-ERK, PI3K-AKT, and STAT3, which are essential for OS cell proliferation, invasion, differentiation, and drug resistance. In recent years, significant progress has been made in the development of small-molecule inhibitors and specific antibodies targeting MET for OS therapy. The use of combination therapy as a treatment strategy involves the use of MET inhibitors in conjunction with chemotherapy, immunotherapy, and other targeted therapies. This approach has the potential to overcome resistance and improve therapeutic efficacy. This review summarises the mechanisms of MET signalling in OS, with a focus on the progress of MET-targeted therapies and their combination with other therapeutic strategies. The study provides valuable insights into future research directions, offering novel perspectives on the role of MET as a therapeutic target in OS.

## 1 Introduction

Osteosarcoma (OS) is the most prevalent primary malignant bone tumour, arising from malignant mesenchymal cells that produce immature bone or osteoid. It predominantly affects adolescents, and its precise etiology remains unclear (1). The prognosis for OS is often poor due to its tendency to metastasize to the lungs and other organs, with the 5-year survival rate for metastatic OS estimated at approximately 20%–30% (2). Prior to metastasis, treatment mainly relies on surgical intervention, such as wide *en bloc* resection or amputation, to prolong patient survival, although these procedures significantly compromise quality of life. In recent years, the combination of novel chemotherapy regimens with surgical approaches has led to improved survival in some patients, but more than 80% still die from lung metastases owing to the highly metastatic nature of the disease (3–5). Consequently, there is an urgent need to identify novel therapeutic targets to improve survival outcomes in patients with metastatic or treatment-resistant OS.

MET, also known as c-MET (6), is a receptor tyrosine kinase proto-oncogene. Its nomenclature is frequently misinterpreted as “mesenchymal-epithelial transition factor”; however, the term “MET” actually derives from its discovery in 1984 by Cooper et al., who identified the gene through experiments involving human osteosarcoma (HOS) cells treated with the chemical mutagen N-methyl-N′-nitro-N-nitrosoguanidine (MNNG). Oncogenic transformation was observed after transfecting the extracted DNA into NIH 3T3 mouse fibroblasts, leading to the naming of the gene as MET (7). In 1985, Dean et al. sequenced the MET gene and found that its protein product shares high homology with members of the tyrosine kinase family, indicating structural features typical of receptor-type kinases (8). This discovery laid the groundwork for the subsequent identification of MET as the high-affinity receptor for hepatocyte growth factor (HGF). HGF was first described by Nakamura et al. in 1984, following its isolation from the serum of partially hepatectomized rats. Initially termed “hepatotropin,” it was identified as a novel mitogen capable of stimulating DNA synthesis in primary hepatocytes (9). Concurrently, Stoker et al. discovered the same protein as a growth factor that could induce epithelial cell scattering and migration, and designated it “scatter factor”, thereby emphasising its critical role in regulating cell motility and tissue morphogenesis (10). In 1991, Bottaro et al. (11) provided the first definitive evidence that MET functions as the high-affinity receptor for HGF. Using radiolabeled ligand cross-linking and immunoprecipitation assays, they confirmed the direct interaction between HGF and MET and demonstrated that ligand binding triggers receptor activation and downstream tyrosine kinase signaling, thereby establishing the molecular basis of the HGF/MET signaling axis. The HGF/MET pathway plays a key role in cell migration, proliferation, and invasive growth, and is aberrantly activated in various malignancies (12–15). In patients with OS, MET is frequently overexpressed, and such overexpression is closely associated with poor clinical outcomes (16). Studies have demonstrated that sustained activation and upregulation of MET can drive the malignant transformation of osteoblasts into OS cells, thereby promoting tumor initiation and progression (17). This review aims to elucidate the molecular mechanisms by which the MET signaling pathway contributes to the pathogenesis of OS and to summarize recent advances in MET-targeted therapies and combination treatment strategies, with the goal of informing clinical decision-making and advancing precision oncology in OS.

## 2 Biological mechanisms of the HGF/MET pathway

### 2.1 The role of the MET receptor and its ligand HGF

The MET receptor and its ligand, HGF, have been shown to play critical roles in the initiation and progression of various malignancies. Studies have demonstrated that aberrant overexpression of HGF is closely associated with poor prognosis in several types of cancer, including gastric and lung cancers (18–21), Furthermore, persistent and excessive activation of the MET/HGF signalling pathway frequently enhances the invasive and metastatic potential of tumour cells. Furthermore, mutations in the MET receptor have been demonstrated to impact its binding affinity for HGF and the efficiency of downstream signal transduction, in addition to altering the activation patterns of downstream pathways. These alterations have the potential to influence the sensitivity to MET-targeted agents, thereby further increasing the complexity of treatment and impacting therapeutic efficacy. Although all of these mutations occur within the MET gene, their mechanisms of action differ substantially. For instance, Shieh et al. (22) reported that the N375S point mutation, located in the SEMA domain of the MET receptor, significantly reduces ligand-binding affinity by disrupting its interaction with HGF. In contrast, Frampton et al. (23) demonstrated that exon 14 skipping mutations eliminate the ubiquitination site (e.g.,.Y1003), thereby impairing receptor degradation and leading to sustained MET activation. This alteration has been associated with persistent activation of downstream pathways, including AKT and ERK, and increased clinical sensitivity to MET inhibitors such as crizotinib. Collectively, these findings highlight the functional heterogeneity of MET mutations and their critical implications for therapeutic response in targeted cancer therapy.

### 2.2 MET signal transduction and its role in the tumour microenvironment

**FIGURE 1:**
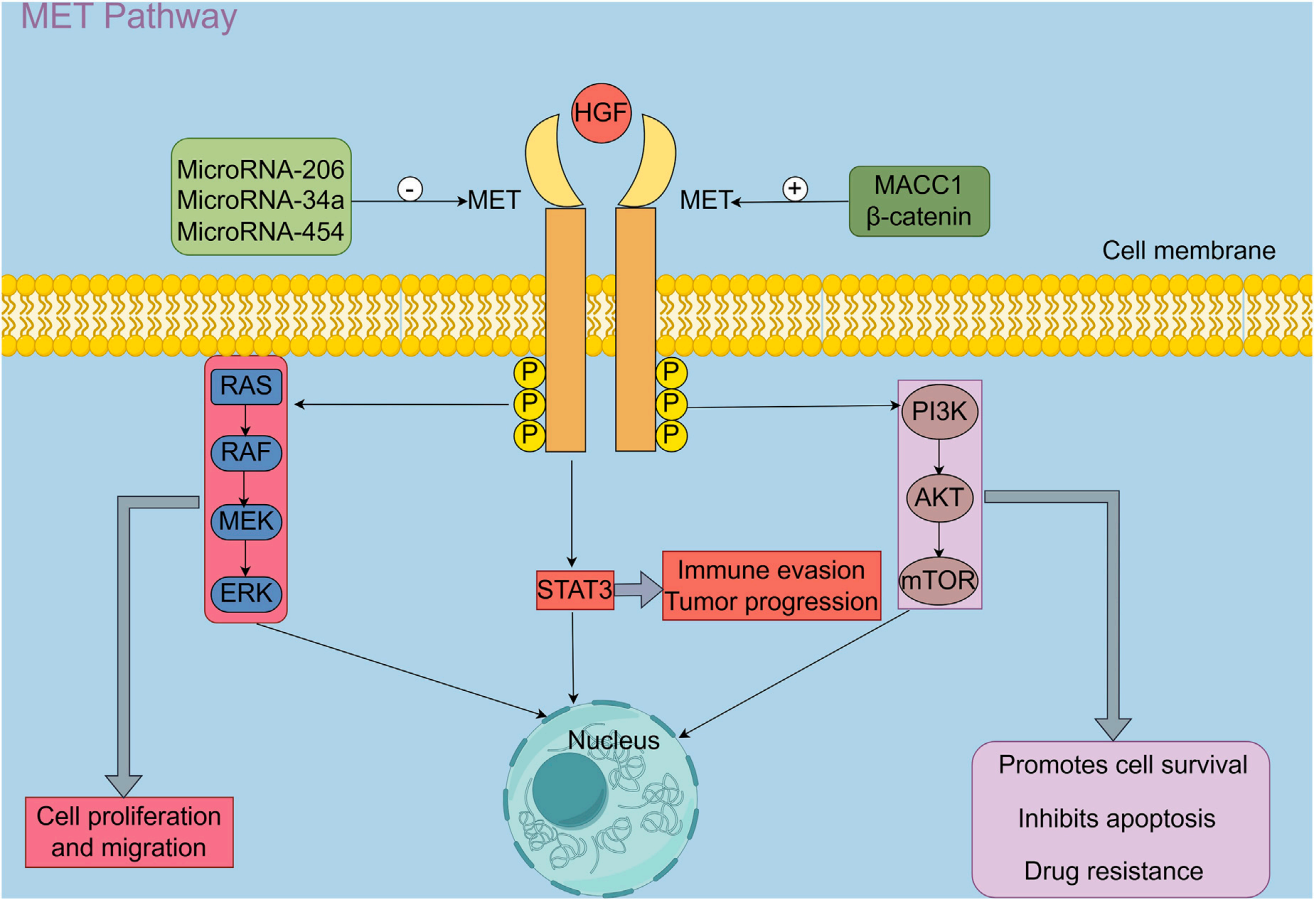
HGF, Hepatocyte growth factor; MET, A receptor tyrosine kinase proto-oncogene; P: Phosphorylation; STAT3, Signal transducer activator transcription factor 3; RAS/RAF/MEK/ERK pathway, Rat sarcoma virus (RAS)/Rapidly accelerated fibrosarcoma (RAF)/Mitogen-activated protein kinase kinase(MEK)/Extracellular signal-regulated kinase(ERK) signaling pathway; PI3K/AKT/mTOR pathway, Phosphoinositide 3-kinase (PI3K)/AKT/Mammalian target of rapamycin (mTOR); β-catenin, β-catenin is a core transcriptional regulator of the Wnt/β-catenin signaling pathway; MACC1, Metastasis-associated in colon cancer-1 (By Figdraw 2.0).

The intracellular signaling cascade of the MET pathway is initiated by the binding of HGF to the MET receptor, which triggers receptor dimerization and activation of its intrinsic tyrosine kinase activity (Figure 1). Activated MET undergoes autophosphorylation on multiple tyrosine residues, creating docking sites for adaptor and scaffold proteins such as GAB1. This leads to the recruitment and activation of several downstream signaling pathways, including the RAS–MAPK, PI3K–AKT–mTOR, STAT3, NF-κB, and JNK/p38 MAPK cascades. These pathways function cooperatively to regulate a broad range of biological processes, including cell proliferation, survival, migration, invasion, and angiogenesis (24). In addition to promoting tumour progression through enhanced cell proliferation and migration, the HGF/MET pathway also modulates the function and infiltration of immune cells within the tumour microenvironment, thus contributing to immune evasion (21). Shen et al. (25) reported that activation of the HGF/c-MET pathway significantly enhances several immune-related signalling pathways, including cytokine–receptor interactions, chemokine signalling, T cell activation, and natural killer (NK) cell-mediated cytotoxicity. These changes influence the infiltration and functional states of immune cells such as macrophages, dendritic cells, and regulatory T cells (Tregs), leading to a reprogramming of the tumour immune microenvironment and ultimately promoting tumour invasiveness and metastatic potential.

Upon binding to its receptor, MET, HGF induces MET autophosphorylation and activates several canonical downstream signalling pathways, including RAS/RAF/MEK/ERK, PI3K/AKT/mTOR, and STAT3. These cascades have been demonstrated to cooperatively regulate a variety of biological processes in tumour cells, including proliferation, migration, immune evasion, and drug resistance (24). Furthermore, a multitude of upstream regulatory factors have been identified as playing critical roles in modulating MET expression. Specifically, microRNA-206, microRNA-34a, and microRNA-454 have been shown to negatively regulate c-MET expression and suppress its activity (26–29). In contrast, MACC1 has been demonstrated to bind directly to the c-MET promoter region, thereby enhancing its transcriptional activity (30, 31). Furthermore, upon activation of the Wnt/β-catenin pathway, β-catenin accumulates in the nucleus and forms transcriptional complexes with TCF/LEF, which in turn promote the transcription of c-MET and other oncogenic targets, thereby enhancing their expression and contributing to malignant tumour progression (32).

## 3 Role of MET signalling in OS progression

The elevated expression and oncogenic function of the MET gene in OS cells has garnered increasing attention. As the receptor for HGF, MET is closely associated with OS initiation, particularly exhibiting marked overexpression in commonly used OS cell lines such as U2OS and 143B (2, 5, 33). This heightened expression has been shown to significantly enhance tumour cell proliferation, as well as promote cell migration and invasion. Patanè et al. (17) demonstrated, using a primary human osteoblast model, that sustained MET overexpression induces malignant transformation, characterised by continuous proliferation, anchorage-independent growth, and tumourigenic phenotypes typical of OS. *In vivo* experiments further revealed that MET-overexpressing cells formed solid tumours resembling human OS in immunodeficient mice, with tumour growth highly dependent on MET signalling. These findings provide direct evidence supporting the critical driver role of MET overexpression in the initiation and progression of OS. Furthermore, Zhan et al. (26) established a miR-206 overexpression model in MG63 and U2OS cells and found that MET downregulation significantly inhibited cell proliferation and promoted apoptosis. Functional rescue experiments, involving the transfection of MET-overexpressing plasmids, confirmed that elevated MET expression activates the PI3K–AKT and MAPK–ERK pathways, significantly enhancing OS cell proliferative capacity. Conversely, treatment of OS cells with the MET inhibitor PHA-665752 markedly suppressed proliferation and increased apoptosis (4). Additionally, a study by Kawano et al. (34) demonstrated that the HSP90 inhibitor 17-DMAG effectively blocked OS cell proliferation and induced apoptosis by inhibiting MET signalling, further supporting the role of MET as a key oncogenic driver in OS cells.

The critical role of MET signalling in the initiation and progression of OS has been extensively investigated, with its aberrant activation implicated in the regulation of OS cell proliferation, migration, and differentiation. Upregulation of the HGF/MET signalling pathway significantly promotes OS cell proliferation, not only by directly modulating cell cycle progression but also by enhancing cell survival through activation of the PI3K/Akt pathway, thereby accelerating tumour growth (35). Moreover, aberrant MET signalling plays a pivotal role in OS invasion and metastasis. High MET expression markedly increases the migratory and invasive capabilities of tumour cells, whereas MET silencing effectively reduces cell motility, limits distant metastasis, and improves patient prognosis (17, 36). MET overexpression has also been shown to enhance the self-renewal capacity of OS cells, which may be closely linked to the activation of downstream pathways such as ERK1/2 (27, 37). In addition, MET signalling contributes to OS cell differentiation by regulating cytoskeletal reorganisation and the expression of cell adhesion molecules, thereby influencing phenotypic transitions associated with increased invasiveness and metastatic potential (16). Consequently, further elucidation of the regulatory mechanisms of MET signalling in OS cell proliferation, migration, and differentiation is essential for understanding its role in OS pathogenesis and for guiding the development of more effective targeted therapeutic strategies.

## 4 Therapeutic targeting of the MET signalling axis in OS

### 4.1 Small-molecule inhibitors targeting MET

In recent years, a growing body of research has focused on the clinical potential of small-molecule inhibitors targeting the MET signalling pathway, particularly in the treatment of malignant solid tumours such as OS. Aberrant activation of MET signalling promotes tumour cell proliferation, migration, and invasion, thereby making targeted inhibition of this pathway a key focus in cancer therapy research (31, 38, 39). Zeng et al. (16) conducted a comprehensive review highlighting the therapeutic relevance of MET in OS and proposed the MET pathway as a novel target for precision oncology. Moreover, studies have shown that MET inhibitors not only effectively suppress OS cell proliferation and migration but also induce apoptosis, reinforcing the rationale for MET-targeted therapy (29). Among these compounds, the c-MET inhibitor PHA-665752 has been shown to significantly inhibit OS cell proliferation and promote apoptosis by suppressing the ERK1/2 signalling pathway. *In vivo* studies further demonstrated that PHA-665752 effectively inhibited tumour growth in OS xenograft mouse models, providing compelling experimental evidence for MET signalling as a promising therapeutic target in OS (16).

In recent years, clinical studies have revealed a strong association between aberrant activation of the MET signalling pathway and tumour drug resistance, underscoring MET as a promising therapeutic target in OS (40). SCR1481B1, a novel selective c-MET small-molecule inhibitor, has been shown to effectively inhibit MET kinase activity and block the sustained activation of its downstream pathways, thereby suppressing OS cell proliferation and migration. Recent investigations have explored the use of SCR1481B1 in combination with the multi-target tyrosine kinase inhibitor (TKI) anlotinib to overcome resistance caused by high MET expression (41). Anlotinib, a novel multi-target TKI, has been demonstrated to significantly suppress OS growth and metastasis through dual inhibition of VEGFR2 and MET (38). Furthermore, studies have indicated that the crosstalk between VEGFR and MET signalling pathways plays a crucial role in tumour angiogenesis and disease progression. The development of dual-target inhibitors that simultaneously block both pathways has thus become an active area of research (42). Additionally, combining MET inhibitors with other therapeutic agents has shown remarkable synergistic effects, particularly in overcoming drug resistance and enhancing treatment efficacy across various tumour types (43, 44).

In summary, the development of small-molecule inhibitors targeting the MET signalling pathway, along with their associated therapeutic strategies, has introduced promising new avenues for the precision treatment of OS. Nonetheless, further studies are required to clarify the underlying mechanisms and to refine optimal clinical application protocols.

### 4.2 Application of c-MET-targeted specific antibody therapies in OS

Targeted antibodies represent a key component of current tumour-directed therapeutic strategies. These include conventional monoclonal antibodies (mAbs) and novel bispecific antibodies (BsAbs). The former act by binding with high affinity to a single antigen, whereas the latter can simultaneously recognize two or more distinct targets, thereby enhancing antitumour efficacy or modulating immune responses. MAbs targeting the MET receptor primarily function by competitively inhibiting the binding of HGF to MET, effectively suppressing downstream signaling and reducing tumour cell proliferation and migration (45–48). In contrast to conventional mAbs, which are limited to single-antigen recognition, BsAbs are capable of targeting two molecular epitopes simultaneously. This offers significant advantages in blocking multiple oncogenic signaling pathways and enhancing immune cell recruitment (49, 50).

Preliminary findings presented in a conference abstract described a novel c-Met monoclonal antibody (LA480). This antibody possesses both neutralising and internalising properties and is capable of inhibiting both HGF-dependent and HGF-independent activation of the MET signalling pathway. The study demonstrated that LA480 significantly reduced the number of c-Met receptors on the cell surface by blocking the interaction between HGF and the c-Met receptor and effectively decreased total and phosphorylated c-Met levels within tumour cells. Collectively, these effects lead to the inhibition of cell proliferation across a range of tumour cell lines. Furthermore, LA480 exhibited potent antitumour activity in both ligand-dependent and ligand-independent xenograft tumour models in mice (51).Although further preclinical and clinical validation is warranted, these findings suggest the potential therapeutic value of mAbs targeting the MET receptor in OS treatment.

OS is a highly aggressive malignancy characterised by a strong propensity for pulmonary metastasis, the most common form of distant spread and a key determinant of prognosis. Targeting the MET signalling pathway with monoclonal antibody-based therapies may offer a novel treatment strategy for patients with lung-metastatic OS. MAbs enhance immune-mediated tumour clearance by mediating antibody-dependent cellular cytotoxicity (ADCC) and complement-dependent cytotoxicity (CDC), thereby improving immune recognition and elimination of OS cells and potentially improving control over pulmonary lesions. MAbs targeting HGF or MET have demonstrated significant antitumour potential in non–small-cell lung cancer (NSCLC). For example, the anti-MET monoclonal antibody MetMAb, when combined with the epidermal growth factor receptor (EGFR) inhibitor erlotinib, significantly improved progression-free survival (PFS) and overall survival in MET-positive NSCLC patients in a phase II clinical trial (52), In addition, Zhuang et al. (48) reported that anti-MET mAbs enhanced the synergistic effects of radiotherapy and EGFR-targeted therapy, further supporting their combinatorial potential. These findings suggest that such strategies may also offer therapeutic benefits for other malignancies with a high risk of pulmonary metastasis, such as OS. Furthermore, the anti-HGF monoclonal antibody Ficlatuzumab demonstrated favourable tolerability in a phase Ib study when combined with the EGFR tyrosine kinase inhibitor gefitinib, and induced partial responses (PR) in a subset of patients, further underscoring its potential clinical value in the treatment of NSCLC (52). Additionally, preclinical studies have demonstrated that inhibition of the HGF/MET pathway suppresses tumour growth in multiple cancer models and, in some cases, overcomes resistance to other anticancer agents (53, 54). Notably, in transgenic mouse models, blockade of the HGF–MET interaction significantly reduced tumour incidence and growth rate (55). Despite the paucity of research in this area, studies in NSCLC have demonstrated the promising antitumour efficacy of mAbs targeting the HGF/MET pathway, particularly against lung metastases. Therefore, it can be hypothesised that these antibodies may exert therapeutic effects in OS, particularly in pulmonary metastatic lesions, thereby offering a potential new treatment option for patients with metastatic disease.

Despite the limited research on mAbs directly targeting MET in OS, engineered BsAbs targeting MET have recently shown promising therapeutic potential in various malignancies. In contrast to conventional mAbs, which bind to a single antigen, BsAbs are designed to simultaneously recognise two distinct molecular targets, thereby enabling enhanced antitumour effects through the concurrent inhibition of multiple signalling pathways or the recruitment of immune effector cells (56). For instance, amivantamab is an approved bispecific antibody that targets both EGFR and MET. It binds to the extracellular domains of both receptors, thereby inhibiting EGFR- and MET-driven oncogenic signaling and promoting receptor internalization and degradation, ultimately exerting antitumour effects (57). Although the application of this approach in OS models remains in the early stages of investigation, the aberrant expression and functional significance of both EGFR and MET in OS suggest that dual-targeting strategies warrant further exploration in this context.

### 4.3 Exploration of combination therapy strategies

Despite their demonstrated efficacy in OS, c-MET-targeted therapies face significant clinical challenges due to limitations associated with monotherapies, including compensatory activation of alternative signalling pathways, the development of drug resistance, and tumour microenvironmental adaptation. Consequently, combination therapy strategies are being increasingly explored as a promising strategy to overcome these obstacles (Table 1).

**TABLE 1:** Summary of MET-related combination therapy and mechanism-supported studies in OS.

Author (Year)	Combination strategy and potential therapeutic value	Key findings
McQueen et al. (2011) (58)	Wnt-mediated regulation of c-MET supports a mechanistic basis for combination therapy	Wnt dysregulation may promote c-MET activation and enhance invasiveness; mechanistic crosstalk supports rationale for Wnt/MET-targeted combination therapy. (Type B)
Niu et al. (2015) (29)	miR-454 targets c-MET and functions as a regulatory axis; provides a mechanistic basis for combination strategy	miR-454 is downregulated in OS and directly targets c-MET to inhibit proliferation and invasion. Functional rescue experiments confirm that c-MET overexpression reverses this effect, supporting the miR-454/c-MET axis as a mechanistic basis for combination therapy. (Type B)
Zhao et al. (2015) (28)	miR-34a prodrug + Doxorubicin	Synergistic anti-proliferative and pro-apoptotic effects in 143B and MG-63 cells (CI < 1); *in vivo*, combination group showed significant tumour suppression and necrosis (P < 0.01). (Type A)
Zhu and Wang et al. (2016) (59)	EGCG + Crizotinib	Combination induced G2/S arrest and apoptosis in MG-63 and U-2OS cells (P < 0.05); significant tumour suppression in nude mice, suggesting synergistic efficacy. (Type A)
Chaiyawat et al. (2017) (60)	Activation of c-MET/VEGFR2 is associated with chemosensitivity, suggesting its potential as a predictive biomarker	Activation of c-MET and VEGFR2 in OS correlates positively with tumor necrosis following cisplatin plus doxorubicin treatment, suggesting their potential as predictive biomarkers and supporting c-MET-based individualized combination strategies. (Type B)
Wen et al. (2020) (31)	MACC1 upregulates c-MET and promotes OS progression, indicating the pathway’s potential for combinatorial targeting	MACC1 knockout downregulates c-MET and its phosphorylation, suppressing OS cell migration, invasion, and microtubule stability. This highlights the critical role of the MACC1/HGF/c-MET axis and provides mechanistic support for combination targeting strategies. (Type B)
Wang et al. (2021) (61)	miR-485-3p targets c-MET to suppress glycolysis and metastasis, suggesting its potential as a regulatory target.	miR-485-3p suppresses glycolysis and invasion in OS by targeting c-MET; rescue experiments confirm the MALAT1/miR-485-3p/c-MET axis as a mechanistic basis for potential combination strategies. (Type B)
Meng et al. (2024) (62)	BMS-794833 + Anlotinib	Combination significantly inhibited tumour growth in U2OS xenografts (P < 0.001); *in vitro* studies confirmed synergistic mechanisms. (Type A)
Wang et al. (2024) (41)	SCR1481B1 combined with anlotinib, delivered via microrobotic system	TARGET analysis linked co-expression of VEGFR2 and MET to poor prognosis; *in vitro*, triple combination showed strong synergy (CI < 0.3), but no *in vivo* data reported. (Type A)
Fu et al. (2025) (63)	MET PROTAC degrader + small-molecule MET inhibitor	Significant tumor inhibition and bone destruction alleviation were observed in the MNNG/HOS nude mouse model (P < 0.001); induced NCL-MDM2–dependent ubiquitination and degradation of c-MET *in vitro*. (Type A)

Note, Studies included in this table are classified into two categories.

Type A: Actual combination therapy studies, in which two or more therapeutic agents (e.g., MET inhibitors and chemotherapeutics) were co-administered and experimentally evaluated for therapeutic efficacy.

Type B: Mechanism-supported or predictive biomarker studies, in which c-MET plays a functional role in modulating treatment response (e.g., via miRNA regulation or phosphorylation status), although no pharmacological co-treatment was conducted. These entries provide mechanistic or predictive rationale for future combination therapy strategies involving c-MET in OS.

• Type A: Actual combination therapy studies, in which two or more therapeutic agents (e.g., MET inhibitors and chemotherapeutics) were co-administered and experimentally evaluated for therapeutic efficacy.
• Type B: Mechanism-supported or predictive biomarker studies, in which c-MET plays a functional role in modulating treatment response (e.g., via miRNA regulation or phosphorylation status), although no pharmacological co-treatment was conducted. These entries provide mechanistic or predictive rationale for future combination therapy strategies involving c-MET in OS.

The advent of non-coding RNAs has expanded the scope of intervention for combination therapies. Niu et al. (29) found that the microRNA (miRNA) miR-454 is downregulated in OS, and that it directly targets the 3′untranslated region (3′-UTR) of c-MET mRNA, thereby suppressing c-MET protein expression. The negative regulatory effect of miR-454 on c-MET suggests the potential for a synergistic antitumour effect in OS—particularly in inhibiting cell proliferation and invasion—when combining strategies to upregulate miR-454 expression (e.g., via miRNA mimics or delivery systems) with c-MET inhibitors. This approach warrants further investigation. In a similar study, Wang et al. (61) demonstrated that miR-485-3p can directly inhibit both c-MET and AKT3, while the long non-coding RNA (lncRNA) MALAT1 reverses this effect through a competitive endogenous RNA (ceRNA) “sponging” mechanism, thereby activating the downstream AKT/mTOR pathway. Consequently, these findings suggest that modulation of the MALAT1/miR-485-3p/c-MET axis may serve as an important adjunct strategy to c-MET–targeted therapy. In another study, Zhao et al. (28) developed a miR-34a prodrug, which, when co-administered with doxorubicin, synergistically downregulated the expression of c-MET, CDK6, and SIRT1, significantly enhancing antitumour efficacy while reducing doxorubicin-associated toxicity. These findings support the feasibility of miRNA replacement therapy as a complementary approach in c-MET–based combination treatment strategies.

From an upstream regulatory perspective, Wen et al. (31) demonstrated that MACC1 promotes OS cell proliferation and migration by enhancing c-MET transcriptional activity, suggesting that co-inhibition of MACC1 and c-MET may offer improved therapeutic outcomes, especially in cases with high c-MET expression. Additionally, McQueen et al. (58) reported that the Wnt/β-catenin pathway upregulates c-MET expression and plays a role in metastasis and epithelial–mesenchymal transition (EMT). Inhibitors of the Wnt signalling pathway have shown promising efficacy in OS and other malignancies, particularly in suppressing tumour proliferation, metastasis, drug resistance, and the maintenance of cancer stem cells. Therefore, combining Wnt and c-MET inhibitors may effectively reduce pulmonary metastasis risk and enhance the responsiveness to c-MET–targeted therapies, providing a novel dual-axis strategy for OS treatment.

In the context of chemotherapy-based combination strategies, c-MET inhibitors demonstrate synergistic activity with conventional agents such as doxorubicin and cisplatin. Mechanistically, Chaiyawat et al. (60) identified the activation status of receptor tyrosine kinases (RTKs), including c-MET and VEGFR2, as being closely associated with OS responsiveness to cisplatin and doxorubicin, suggesting their potential as predictive biomarkers for combination therapies. Similarly, In a canine OS model, Marley et al. (64) reported that dasatinib, a multi-target tyrosine kinase inhibitor primarily acting on SRC family kinases, did not fully suppress MET phosphorylation. However, under conditions in which MET activation was induced by HGF, dasatinib was still able to significantly inhibit the migration and invasion of OS cells, suggesting that it may exert antimetastatic effects by interfering with downstream functions of the HGF/MET signaling pathway. Moreover, the combination of dasatinib with postoperative carboplatin chemotherapy demonstrated the potential to prolong survival in dogs with OS, supporting the feasibility of combining MET-targeted inhibitors with conventional chemotherapy. In addition, Sampson et al. (65) reported that the c-MET inhibitor PF-2341066 significantly suppresses OS cell proliferation and metastasis while potentiating the efficacy of chemotherapeutic agents such as doxorubicin and cisplatin.

Beyond conventional chemotherapy combinations, the integration of natural bioactive compounds with targeted therapies has shown promising potential. Zhu and Wang reported that the green tea polyphenol epigallocatechin-3-gallate (EGCG) suppresses OS cell proliferation and induces apoptosis by upregulating miR-1 expression, which in turn downregulates its target gene c-MET. Subsequent experiments showed that combining EGCG with the c-MET inhibitor crizotinib significantly enhanced cell cycle arrest and apoptosis induction, indicating a synergistic antitumour effect. These findings suggest that natural compounds in combination with targeted agents may offer a novel therapeutic strategy for the treatment of OS (59).

To address resistance to MET inhibitors, combination strategies targeting multiple signalling pathways have gained increasing attention. In a seminal study, Tang et al. (39) demonstrated that OS cells acquire resistance to the MET inhibitor PF02341066 via VEGFR2 signalling activation induced by the vascular microenvironment. Co-treatment with cabozantinib effectively reversed this mechanism and significantly enhanced antitumour efficacy. Similarly, Meng et al. (62) proposed a dual-targeted strategy combining the multi-target tyrosine kinase inhibitor (TKI) BMS-794833 with anlotinib to overcome resistance by co-inhibiting the VEGFR/Ras/CDK2 pathways. *In vitro* assays using U2OS and MG63 cell lines—including viability, colony formation, migration, and apoptosis—revealed that the combination therapy significantly outperformed monotherapy across multiple parameters, demonstrating synergistic antitumour effects. *In vivo*, a subcutaneous U2OS xenograft model showed significantly reduced tumour volume and final tumour weight following combination treatment, accompanied by decreased Ki-67 expression and no observable systemic toxicity, confirming both efficacy and safety. Furthermore, Wang et al. (41) developed a magnetically driven hydrogel microrobot platform to enable targeted co-delivery of the MET inhibitor SCR1481B1 and the VEGFR inhibitor anlotinib, enhancing synergistic cytotoxicity within the tumour microenvironment. Their findings revealed that high MET expression in OS cells was closely associated with resistance to anlotinib, while co-administration of SCR1481B1 markedly restored drug sensitivity. This strategy demonstrated strong synergistic effects in both 2D monolayer and 3D organoid models, while effectively reducing systemic toxicity, highlighting its promise as a precision delivery system for combination therapy.

In the domain of immunotherapy, the role of the c-MET signalling pathway in regulating the tumour immune microenvironment has attracted increasing attention. In this context, Fu et al. (63) developed a dual-aptamer-functionalised c-MET PROTAC degrader (AS1411-SL1-2), which not only significantly reduced tumour burden but also demonstrated favourable target selectivity and safety. These findings suggest that combining this strategy with small-molecule c-MET inhibitors may hold therapeutic potential. Furthermore, Glodde et al. (66) found that c-MET signalling promotes the infiltration of immunosuppressive neutrophils. Co-administration of a c-MET inhibitor with anti-PD-1 antibodies has been shown to alleviate immunosuppression, enhance T cell activity, and improve the efficacy of immunotherapy.

In summary, c-MET–centered combination therapy strategies in OS encompass a broad spectrum of interventions, including small-molecule inhibitors, non-coding RNAs, biological regulatory axes, and modulation of the tumour immune microenvironment. These approaches demonstrate improved efficacy and delayed resistance, providing a theoretical and experimental foundation for future personalised treatment strategies. Moving forward, research efforts should focus on optimising target combinations, identifying reliable predictive biomarkers, and conducting clinical validation, thereby advancing combination therapies toward multi-targeted and precision-based approaches.

## 5 Conclusion

With advancing understanding of the pathogenesis of OS, the MET signalling pathway has emerged as a key focus of research due to its pivotal role in tumour initiation, progression, and metastasis. Current studies have confirmed that aberrant activation of MET is closely associated with OS cell proliferation, invasion, metastasis, and drug resistance. Preclinical investigations have demonstrated the potential of targeting the MET pathway—through small-molecule inhibitors, specific antibodies, and combination therapy strategies—to improve therapeutic outcomes. Notably, combination strategies offer a promising approach for overcoming drug resistance, enhancing treatment efficacy, and enabling coordinated multi-pathway intervention, representing a substantial breakthrough in the evolution of OS therapy.

Nevertheless, the clinical translation of MET-targeted therapies in OS continues to face several major challenges, including the precise identification of responsive patient populations, the durability of therapeutic efficacy, safety evaluation, and the complexity of resistance mechanisms. Future research should focus on optimising target combinations from a precision medicine perspective, discovering and validating predictive biomarkers, systematically evaluating combination regimens, and developing animal models and clinical trial designs that better reflect clinical realities. The integration of interdisciplinary technologies—including nanodelivery systems, immune modulation, and synthetic biology—offers new opportunities to revitalise MET-targeted interventions and advance their clinical applicability.

In conclusion, the MET signalling pathway represents a key therapeutic target in OS, with broad prospects for research and clinical translation. Continued advances in basic research and clinical practice are expected to yield more precise, effective, and sustainable treatment options for patients with OS.
